# Equity in distribution of public subsidy for noncommunicable diseases among the elderly in India: an application of benefit incidence analysis

**DOI:** 10.1186/s12889-019-8089-y

**Published:** 2019-12-26

**Authors:** Montu Bose, Somdutta Banerjee

**Affiliations:** 1 Department of Business & Sustainability, TERI School of Advanced Studies, New Delhi, India; 2 Department of Economics, Indian Institute of Foreign Trade, New Delhi, India

**Keywords:** Health equity, Non-communicable diseases, Benefit incidence analysis, Elderly in India

## Abstract

**Background:**

Rapid ageing of the population and increasing non-communicable diseases (NCDs) among the elderly is one of the major public health challenges in India. To achieve the Universal Health Coverage, ever-growing elderly population should have access to needed healthcare, and they should not face any affordability related challenge. As most of the elderly suffers from NCDs and achieving health-equity is a priority, this paper aims to - study the utilization pattern of healthcare services for treatment of NCDs among the elderly; estimate the burden of out-of-pocket expenditure for the treatment of NCDs among the elderly and analyze the extent of equity in distribution of public subsidy for the NCDs among the elderly.

**Methods:**

National Sample Survey data (71st round) has been used for the study. Exploratory data analysis and benefit incidence analysis have been applied to estimate the utilization, out-of-pocket expenditure and distribution of public subsidy among economic classes. Concentration curves and indices are also estimated.

**Results:**

Results show that public-sector hospitalization for NCDs among the elderly has a pro-rich trend in rural India. However, in urban sector, for both inpatient and outpatient care the poorest class has substantial share in utilization of public facilities. Same result is also observed for rural outpatient care. Analysis shows that out-of-pocket expenditure is very high for both medicine and medical care even in public facilities for all economic groups. It is also observed that medicine has the highest share in total medical expenses during treatment of NCDs among the elderly in both the region. Benefit incidence analysis shows that the public subsidy has a pro-rich distribution for inpatient care treatment in both the sectors. In case of outpatient care, subsidy share is the maximum among the richest in the urban sector and in the rural region the poorest class gets the maximum subsidy benefit.

**Conclusions:**

It is evident that a substantial share of the public subsidies is still going to the richer sections for the treatment of NCDs among the elderly. Evidences also suggest that procuring medicines and targeted policies for the elderly are needed to improve utilization and equity in the public healthcare system.

## Background

Rapid ageing of the population, demographic and epidemiological transition along with increasing health inequity and inequalities impose major public health challenges in most of low- and middle-income countries (LMICs) [1–4]. The United Nations (UN) has documented that the population aged 60 years or above is growing at a rate of 3.26% and by 2050 almost all areas except Africa would have nearly 25% of their populations aged 60 or more [5, 6]. The pursuit of health equity in ageing societies raises several concerns – on the one hand there is persisting health inequity among social groups and on the other hand, distribution of scarce public healthcare resources always raises a question on distributive justice. Fair and effective functioning of the public health system is more important and relevant to elderly people as they carry higher burden of diseases, specifically noncommunicable diseases [7]. To achieve Universal Health Coverage (UHC), the World Health Organization (WHO) [8] has identified three aspects of healthcare systems – ensuring that all people receives the needed quality healthcare services, everyone should be protected from health threats and financial hardship due to treatment should be avoided. Additionally, Equity in health system is an international priority. This actually demands assessment of healthcare interventions among socioeconomically disadvantageous sections. However, without considering the healthcare needs of the ever-growing numbers of elder people, UHC would be impossible to achieve [9].

In India, the proportion of population aged 60 years and above is projected to increase from 9% (2015) to 20% by 2050 [5, 6]. This percentage point increase is a remarkable increase in absolute terms. It is projected that the numbers of elderly in India would reach 159 million by 2025 [10] and it is also estimated that by 2050, elderly population would surpass the population of children below 14 years [11]. It is well documented in the literature that the increased burden of non-communicable diseases and subsequent healthcare need have put utmost concerns over the aged populations in India [12]. Literature have also indicated uneven access to healthcare services among the elderly in India for NCDs [13]. Moreover, the resource constraint public healthcare system poses additional challenges to meet the healthcare needs of the elderly people and reducing the socioeconomic inequity and inequalities in health. Analyzing literature on health of the elderly in India, Dey et al., (2012) found that the elderly population does not have access to needed healthcare and those who have physical access to healthcare services, many of them, face affordability related challenges due to cost of accessing the healthcare [13].

There are several studies available on NCDs and related utilization of healthcare services and costs of the aged in the Indian context. Using NSS (2004) data Agrawal and Keshri (2014) described the demographic transition in India and health seeking behavior of the aged widows [14]. It is documented in the paper that morbidity prevalence is higher among the aged and share of NCDs are substantially higher than the communicable diseases. Analyzing the same data Joe et al., (2015) shows the socioeconomic inequality in utilization of healthcare services among the elderly [15]. The study also demonstrates the pro-rich distribution of utilization of healthcare services. Kastor and Mohanty (2018) analyzed nationally representative data collected during 1995 and 2014 to show the changes in hospitalization pattern and associated costs [16]. It has been revealed from the study that the hospitalization rate has been more than doubled during this time period and this is primarily due to increased hospitalization among infant and aged members. Substantial increase in out-of-pocket expenditure among the aged households is also evident from recent studies [16–19]. Analyzing NSS data on outpatient care services in Kerala, Mukherjee and Levesque (2012) demonstrates the inequity in utilization of outpatient care services between poor and non-poor aged patients [20]. Few studies have also analyzed NSS data to study the economic burden of specific NCD ailment in India and its adverse consequences on the households [21–24]. However, there is dearth of literature on the extent of equity in distribution of public subsidies among the aged in India.

Given this background, the paper attempts to study the extent of access to healthcare services and equity in distribution of public subsidy among the elderly who are suffering from noncommunicable diseases in India. Specifically, the objectives of the paper are - to study the utilization pattern of healthcare services for treatment of NCDs among the elderly in India, to estimate the burden of out-of-pocket expenditure (OOPE) for the treatment of NCDs among the elderly and to analyze the extent of equity in distribution of public subsidy for the NCDs among the elderly people.

## Methods

### Data

National Sample Survey (NSS) 71st round unit level data on Social Consumption: Health (2014) has been used for the study. This is the most recent data available in India on morbidity, healthcare utilization and related out-of-pocket expenditure (OOPE). NSS adopted stratified multistage design to collect data. The census villages (in the rural sector) and urban blocks (in the urban sector) were considered as the first stage unit (FSU) and the ultimate stage units (USU) were the households. Considering Census 2011 population, the sample villages were selected by probability proportion to size with replacement (PPSWR) to form the FSUs. On the other hand, in the urban sector, number of households of the urban frame survey (UFS) blocks has been used to form the FSUs following the PPSWR method^1^ [25]. The data has been collected from 3,33,104 individuals living in 65,932 households. It is a nationally representative survey covering all states and Union Territories (UTs). Overall, 8.18% of the total sample are in the age group of 60 year and above. The share of elderly is 7.92% in the rural sector and the corresponding figure for the urban sector is 8.52%. Whereas, 17% of the total hospitalized and 28% of the total outpatient visits are recorded by the aged members. NSS collects information on household level as well as individual level characteristics. It records the details of morbidity, hospitalization and corresponding OOPE for doctor’s consultation, diagnostic tests, medicine, transport and other related costs. It has to be noted here that, NSS records the self-reported morbidity and hospitalization information in the survey. The reference period for inpatient care was 365 days and for outpatient care it was 15 days.^2^ Sources of finance for treatment and insurance coverage information is also available in the data. Sample weights are reported for each household and individual in the data. This survey weights have been applied to scale up the estimates at the population level.

### Disease group & MPCE class formation

Following World Development Report (1993) [26] we have cross classified the ailments into three broad categories – Communicable (CD), Noncommunicable (NCD) and other diseases (OD). The classified data has been analyzed to calculate the utilization of healthcare services for inpatient and outpatient care of the aged people suffering from NCDs. NSS also reports usual^3^ monthly expenditure of the households. Following Cain et.al., (2010) and Srivastava et.al., (2016) [27, 28], the Organization for Economic Co-operation and Development (OECD) equivalence scale has been applied to construct the monthly per capita expenditure (MPCE) class from the household expenditure [27]. Specifically, we have divided the total monthly expenditure of a household (T) by the square-root of the household size (N) to get the MPCE^4^ (= T/√N). Then the MPCE has been arranged in ascending order and grouped into four quarters – Poorest (P), Lower Middle (LM), Upper Middle (UM) and Richest (R). However, cost of living largely varies across states and the sectors (rural and urban) within the states. To accommodate these differences, MPCE classes have been formed separately for each state^5^ and sector.

### Out-of-pocket expenditure estimation

The OOPE has been reported under various heads in the NSS data separately for inpatient and outpatient care. This information has been used to calculate the OOPE on medicine and medical care.^6^ OOPE for transport and other non-medical services like food, expenditure on escorts, lodging charges etc. has been added with the medical expenditure to get the total OOPE. In case of inpatient care, OOPE has been reported for each hospitalization episode separately. However, for the outpatient care, NSS reports the total OOPE under various heads for all the outpatient visits together (for multiple visits within the reference period). Therefore, for the present paper we have considered only those aged individuals who have reported noncommunicable diseases related outpatient care utilization in all the visits.

### Benefit incidence analysis

Benefit incidence analysis (BIA) is a method generally applied in the literature to study the extent of equity in any public system. It has both the horizontal^7^ and the vertical equity dimensions [28, 30]. It has to be mentioned here that BIA has been applied in various studies to examine the extent of equity in public subsidy distribution across socio-economic classes. Few such studies are in the Indian context and based on NSS data. A recent study on utilization and benefit both at the national and state level, used NSS and showed that utilization of inpatient and delivery services are pro-poor. It is also revealed that in most of the states out-patient services is also pro-poor. However, users of public healthcare facilities are forced to spend considerable amount as out-of-pocket to supplement the government services [31]. Another study by Mahal et al., (2001) showed the inequity in subsidy distribution across income groups in India for various healthcare services using BIA [32]. Chakraborty (2012), Acharya (2011) and Ngangbam (2015) also applied BIA to study the extent of equity in distribution of public subsidies in the healthcare sector [33–35]. However, there is dearth of literature with a focus on equity in distribution of public subsidy for NCD related treatment among the elderly in India. In this context, applying BIA, the present study attempts to through some light in this area.

According to the literature, benefit incidence is the net government subsidy weighted by the utilization rate [36, 37]. Mathematically, the benefit incidence could be estimated by the formula –

$$ {\uppi}_{\mathrm{j}}=\sum \limits_{k=1}^n{\upalpha}_{\mathrm{i}\mathrm{j}}\frac{\upgamma_{\mathrm{i}\mathrm{k}}}{\upalpha_{\mathrm{i}}}=\sum \limits_{k=1}^n{\updelta}_{\mathrm{i}\mathrm{j}}{\upgamma}_{\mathrm{i}\mathrm{k}} $$

Where,

π_j_ = Benefit of public subsidy enjoyed by group j (here MPCE class); α_ij_ = utilization of service i (here inpatient and outpatient care for NCDs) by group j; k = number of individual; α_i_ = utilization of service i by all groups together; γ_ik_ = government’s net expenditure on individual k for service i and δ_ij_ = group j’s share of utilization of service i.

Specifically, α_ij_ is number of aged people from a particular MPCE class (j) who are suffering from NCDs and utilizing the public healthcare facilities (forms the numerator). Total number of aged people suffering from NCDs and utilizing public facilities for treatment in all the MPCE classes together forms the α_i_ (the denominator). The ratio of α_ij_ and α_i_ is the utilization rate. To calculate the net government subsidy, we have applied the methodologies available from the literature [28, 38–40]. In short, the net subsidy is the difference between the actual cost of providing a service and the user charges. The user charge for utilizing public facilities is available from the NSS data. However, the major challenge is to get the actual cost of providing the service in the public facilities. Following Bose 2018, Srivastava et.al., 2016; Bose & Dutta 2015; Bose 2014 [28, 38–40], we have considered the modal OOPE of the private hospitals as the proxy for actual cost of providing the services in the public hospitals. To accommodate the differences in quality of care, severity of illness and cost of providing the services, state, sector, MPCE class and duration of stay in hospital (for inpatients only)/ total duration of ailment (for outpatient only) have been considered during calculation of modal OOPE in the private hospitals. The assumption we have made here that the OOPE for utilization of healthcare services are the actual cost of the government to provide the service in the public hospitals. However, it is indeed true that the cost of providing services in public and private sectors may vary. But, reason behind choosing the proxy could be justified from different angles. Primarily, there are various types of private healthcare facilities available for treatment and the prices largely varies based on the regions of operation, quality of services etc. To accommodate the differences and to normalize the variation in prices, analysis has been made using large unit level dataset. Additionally, we have tried to reduce the variation in prices, if persists, by analyzing the data separately for state, sector, MPCE class and duration of stay in hospital (for inpatients only)/ total duration of ailment (for outpatient only). On the other hand, the primary objective of benefit incidence analysis is to study the relative position of various socio-economic groups in access and benefit of public subsidy distribution [30, 37]. Therefore, some level of under or over estimation of public subsidy benefit would not affect the relative position of the MPCE groups in the subsidy ladder. Finally, private sector is the only option remains after the public facilities for treatment. Therefore, the difference between the expenditure in public and private facilities for treatment would be the best available proxy for the shadow price of subsidy in public facilities. OOPE for each hospitalization episode (or outpatient visit) has been subtracted from the corresponding modal private OOPE to calculate the net subsidy. Then the net subsidy is weighted by the utilization rate to get the subsidy benefit of the individual.^8^ We have added all the individual subsidy of a particular MPCE class to get the MPCE group specific subsidy. Share of each MPCE class in total subsidy benefit is the estimate of benefit incidence.^9^ Following O’Donnell et al., (2008) and O’Donnell et al., (2016) we have also calculated the concentration index and curves [41, 42]. Concentration curve could be used as a measure of inequality. Specifically, concentration curve could be used to assess the public subsidies are targeted towards the poor or not [42]. To derive the concentration curve for health inequality, we need two variables – (a) the health variable (here the public subsidy), the distribution of which are subject of interest and (b) a socio-economic variable (here MPCE class) against which the distribution is to be assessed. Concentration index on the other hand, represents twice the area between the concentration curve and the line of equality (45^0^ line). The index takes the negative value when the curve lies above 45^0^ line and vice versa.

## Results

The results of the paper have been represented under three subsections – utilization of healthcare services, OOPE for the treatment of NCDs and the benefit incidence of public subsidy.

### Utilization

Ailment group wise utilization of healthcare services for inpatient (IP) and outpatient (OP) care by the elderly people has been reported in Table 1. It is observed that 42% of the total hospitalization and 54% of the total OP visits are for NCDs in India among the elderly. Additionally, 46% of the total hospitalization are for NCD-related treatment in the urban sector and the corresponding figure for the rural sector is 40%.

**Table 1:** Disease, MPCE Class and Sector wise Utilization of Healthcare Services in India (in %)

Category	Groups	Inpatient			Outpatient		
		Rural	Urban	Combine	Rural	Urban	Combine
	Ailment Group						
Utilization of Healthcare Facilities	CD	19.57	20.97	20.03	39.70	32.20	36.90
	NCD	39.78	46.14	41.87	50.25	60.46	54.05
	OD^a^	40.65	32.90	38.11	10.05	7.34	9.04
	MPCE Class						
Utilization of Healthcare for NCDs	Poorest	16.75	18.80	17.49	19.51	20.18	19.79
	Lower Middle	19.54	23.04	20.80	22.83	21.35	22.22
	Upper Middle	25.02	27.55	25.93	25.64	26.14	25.85
	Richest	38.69	30.60	35.77	32.02	32.33	32.15
Utilization of Public Hospitals for NCDs	Poorest	21.92	28.09	23.87	26.94	32.07	28.82
	Lower Middle	24.65	25.90	25.04	23.22	24.96	23.86
	Upper Middle	25.39	28.36	26.33	25.50	21.47	24.02
	Richest	28.03	17.65	24.74	24.34	21.50	23.30

Source: Authors’ estimation based on NSS data (2014)

^a^Other diseases also include various health conditions like pregnancy, illness in the newborn etc. with fever, body aches and the like diseases which cannot be classified as NCD or CD.

Share of NCDs in total OP visit among the elderly is around 60% in the urban sector. Around 50% of all the OP visits in the rural India are for NCDs. Further bifurcating the IP and OP utilization across MPCE classes, we could observe that there is a positive relationship between MPCE class and utilization of healthcare services for treatment of NCDs among the elderly in India. As we move from the poorest to the richest class utilization of healthcare services increases both for IP and OP care.

Similar trend is observed in both the sectors. The highest utilization of IP care in the rural sector is among the richest (39%) followed by the UM (25%) and LM (20%) class. Corresponding percentages for the urban sector are 31%, 28% and 23% respectively. Lowest utilization of IP care is recorded by the poorest class (17%) of the rural sector followed by the urban-poorest (19%) class. Richest class of the rural and urban sector utilizes the maximum OP care followed by the UM class. Poorest class of both the regions has the lowest access to OP visits in India. Further analyzing the data to check the utilization pattern of public facilities for the treatment of NCDs among the elderly, we could notice that the richest class of the rural sector has the highest utilization of public healthcare facilities for the IP care and the poorest class has the lowest utilization.

On the other hand, in the urban sector UM class has the highest utilization of public IP care followed by the poorest class. Interestingly, utilization of public facilities for the OP care is the maximum for the poorest class in both the sectors in India. The lowest utilization of public facilities is observed among the LM and UM class for the rural and urban sector respectively.

### Out-of-pocket expenditure

Table 2 reports the MPCE class wise OOPE for public and private healthcare facilities in India. Three sets of estimates have been made separately for IP and OP care – medicine, medical and total OOPE. It is observed that the total OOPE in the private facilities is around 1.5–2 times high in the rural sector and it is around 2–4 times high in the urban sector compared to the public facilities. This trend is uniformly observed in both the IP and OP care. For both IP and OP care, poorest class is spending the lowest amount to purchase medicine during utilization of public institutions and as we move towards the richest class the medicine-OOPE increases. The same pattern is observed in both the sectors. However, the medical and total OOPE for the poorest class is higher than the LM class for public sector hospitalization in the rural sector. It is clear from the table that medicine is an important component in the total medical expenses for both IP and OP care in public facilities.

**Table 2:** MPCE Class wise Average Out-of-pocket Expenditure for NCD-related Inpatient and Outpatient Care in India (in INR)

Service	MPCE Class	Rural						Urban					
		Public			Private			Public			Private		
		Medicine	Medical	Total	Medicine	Medical	Total	Medicine	Medical	Total	Medicine	Medical	Total
Inpatient	Poorest	2302	5343	7130	4466	17,364	19,245	1980	4722	6047	5375	27,415	29,607
	Lower Middle	2591	4857	6612	5622	20,570	22,860	3441	7300	9235	6482	26,237	28,923
	Upper Middle	4595	9263	11,366	5903	26,990	29,610	3693	9470	11,696	6283	40,218	43,826
	Richest	4017	12,150	15,223	8911	39,363	43,129	5990	18,554	21,479	10,610	64,797	69,239
	All	**3508**	**8260**	**10,487**	**7021**	**30,175**	**33,157**	**3789**	**10,072**	**12,183**	**8100**	**46,976**	**50,614**
Outpatient	Poorest	188	240	294	331	502	591	108	135	186	367	514	565
	Lower Middle	224	296	379	335	454	519	262	354	417	594	863	934
	Upper Middle	372	441	538	475	612	676	352	408	465	501	737	807
	Richest	364	454	549	530	724	833	348	463	524	716	992	1080
	All	**294**	**366**	**449**	**453**	**616**	**703**	**270**	**344**	**401**	**588**	**834**	**908**

Source: Authors’ estimation based on NSS data (2014)

In case of IP care, the medicine expenditure ranges from 32 to 53% of the total medical expenses. Interestingly, the share of medicine in total medical expenditure is around 80% for public sector OP visits. In both the sectors, the highest medicine share is recorded by the LM class for IP care and UM class for OP visits. Surprisingly, the poorest class is also spending a substantial proportion of their total medical cost to purchase medicine during public sector hospitalization or OP visit.

The above result then raises a question that who is enjoying the benefit of the public subsidy. The NSS data has been analyzed and the MPCE class wise share of subsidy benefit for IP and OP care has been calculated and reported in Table 3. It is observed that the distribution of public subsidy for IP care is pro rich and the maximum subsidy share is recorded by the richest class and the poorest class has the minimum share. It is also observed that the poorest class has the minimum share in total subsidy for inpatient care in the rural sector followed by the UM and the LM class. The richest class of both the regions are benefited the maximum from the public subsidy during their hospitalization. In the urban sector, on the other hand, the lowest subsidy share is recorded by the LM class followed by the poorest class.

**Table 3:** MPCE Class wise Benefit Incidence for Inpatient & Outpatient care in India (in %)

MPCE Class	Inpatient			Outpatient		
	Rural	Urban	Combine	Rural	Urban	Combine
Poorest	9.90	21.05	16.09	40.61	18.28	28.55
Lower Middle	27.84	11.69	18.88	16.24	26.81	21.95
Upper Middle	26.63	30.15	28.58	15.57	17.32	16.51
Richest	35.63	37.11	36.45	27.59	37.59	32.99

Source: Authors’ estimation based on NSS data (2014)

**Fig. 1:**
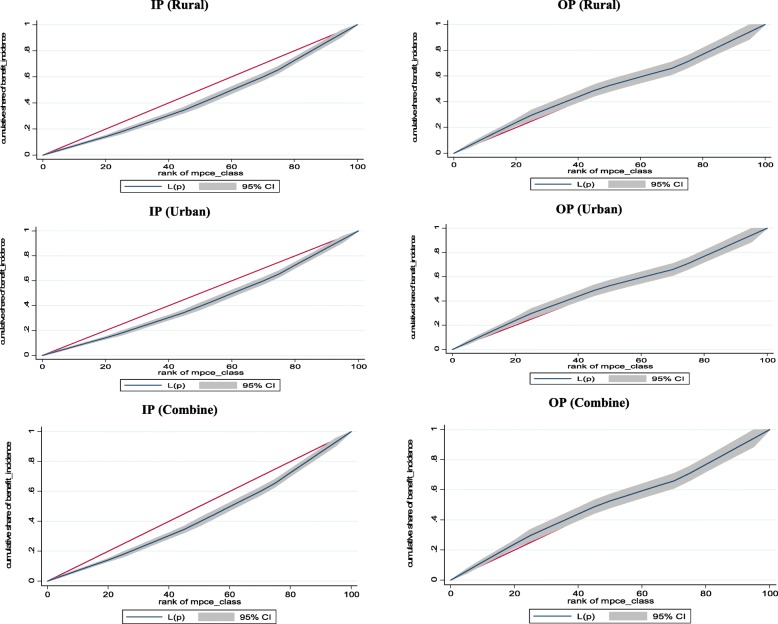
Concentration Curves of the Benefit Incidence of Public Subsidies for IP & OP Care (Source: Authors’ estimation)

Interestingly, there is no specific trend in distribution of public subsidy for OP care. However, the richest class are benefited the maximum from public subsidy followed by the poorest class. The lowest subsidy is observed for the UM class. Sector wise bifurcation of the benefit incidence shows that the poorest class of the rural sector has the maximum benefit share followed by the richest class for OP care. On the other hand, benefit share of the richest class is the maximum followed by the LM class in the urban sector. For better understanding of the distribution of the subsidy, we have derived the concentration curves and presented in Fig. 1.

The distribution of public subsidy is clearly showing a pro-rich trend as the subsidy benefit distribution line lies below the equal distribution line for the IP care in both the sectors. We have also represented the 95% confidence interval for each benefit distribution curve (grey shaded area). Analyzing the distribution of public subsidy for the OP care, we could observe that the benefit distribution line has crossed and merged with the equal distribution line. Therefore, it is very difficult to comment on the distribution pattern of the subsidy benefit from the graphs. To know more about the distribution pattern of the public subsidy we have calculated the concentration indices. The sign of the concentration index indicates the direction of the public subsidy – pro-rich or pro-poor and the magnitude of the index reflects the strength of variability.

The concentration indices are presented in Table 4. It has to be noted here that a positive value of the index denotes a pro-rich distribution of subsidy benefit and a negative value signifies pro-poor distribution. It is observed that the distribution of subsidy benefit is pro-rich for IP care and pro-poor for OP care. Specifically, for both the sectors pro-rich distribution is observed for the IP care and for the OP care the distribution is pro-poor in the rural sector. However, it is observed from the above table that the indices of the urban sector are not statistically significant. Therefore, it is very difficult to comment on the result of the urban sector. On the other hand, the estimates of the rural sectors are highly significant for the IP and OP care. Therefore, significant level of inequity in distribution of public subsidy is evident from the result in case of IP care in the rural region.

**Table 4:** Concentration Index of Benefit Incidence for IP & OP Care

Service	Sector	No. of Observations	Index Value	Robust Std. Error	*p*-value
IP	Rural	1526	0.171	0.043	0.001
	Urban	1271	0.119	0.080	0.152
	Combine	2797	0.141	0.053	0.013
OP	Rural	709	−0.211	0.107	0.061
	Urban	588	0.057	0.096	0.557
	Combine	1297	−0.125	0.085	0.158

Source: Authors’ estimation based on NSS data (2014)

## Discussion

The present paper has analyzed NSS (2014) unit level data on Social Consumption: Health to study the utilization pattern of healthcare services for treatment of NCDs among the elderly in India. The paper also attempts to analyze the burden of OOPE and the extent of equity in distribution of public subsidy among the elderly for NCD-related treatments. It is emerged from the analysis that the prevalence of NCDs among the elderly is very high in India. Hospitalization and OP visits for the NCD related treatment is the highest among all ailment groups for the elderly. Overall utilization of healthcare services shows a pro-rich distribution for both IP and OP care and in both the sectors in India. Utilization of public IP care services has a pro-rich trend in the rural sector. However, the utilization of public facilities for IP care in the urban sector and the OP visits in both the sector have no specific trend. Interestingly, the poorest class have a substantial share in total utilization of public healthcare facilities for these services in the rural and urban sector. The OOPE on medicine is very high among all MPCE groups both for IP and OP care. It is also observed that the OOPE on medicine takes a substantial share of the total medical expenditure even in the public facilities. Further analyzing the data, it has emerged that the richest class gets the maximum subsidy for IP care in both the sectors. However, the poorest class of the rural sector and the richest class of the urban sector records the maximum subsidy share for OP visits. The study has multiple implications and the discussion section analyzes the implications and compare the findings with other similar studies. This section also proposes possible causes and consequences of the findings.

Although there is no specific study available on benefit incidence of public subsidy for NCD related care among the elderly in India, however, few studies have implemented BIA to examine the distribution of public subsidies across socioeconomic groups. Using NSS 52nd round data Mahal et al., (2001) showed the state level variations in utilization of public healthcare services [32]. Their results indicate that for few states the utilization of public healthcare services is pro-poor; however, for many states the distribution is skewed towards the rich. A recent study by Bowser et al., (2019) [31] show that the public outpatient and delivery care is pro-poor in India. The study also documented the state level variations in benefit distribution among the MPCE classes for inpatient and delivery services. Bose and Dutta (2018) [38] analyzed the NSS 71st round data to examine the effectiveness of health financing strategies in three Indian States – Tamil Nadu, Rajasthan and West Bengal. Their results show a pro-poor distribution of public subsidy for IP care. The study also documented the success of medicine distribution scheme in Tamil Nadu and Rajasthan. It is also revealed from our study that medicine has a substantial share in total medical expenditure; specifically, in OP care. If we compare the utilization of public facilities and the benefit share across MPCE groups, we could observe that there is a direct relationship between the two parameters for rural IP care. This actually indicates the barriers in access to IP care facilities in the region. Hospitalization episode not only put direct financial burden on the households; the OOPE burden of it also indirectly pushes the households towards catastrophe and impoverishment through wage loss [43]. It has been observed from the NSS data that most of the Poorest elderly people in the rural sector are illiterate and they are not financially dependent on others (see Table 5 in Appendix). This actually indicates that most of the poorest elderly people are working in the informal sector and wage loss due to hospitalization puts double burden on the households. Literature has also documented most of the aged people are casual or self-employed informal worker who are not entitled to formal retirement benefits and have very low ability to afford healthcare expenses. Consequently, they face paradoxical challenges of remaining both healthy and employed in old age [44]. Interestingly, in the urban sector, the richest class has the lowest utilization share for IP care; however, the benefit share is the maximum for the class. It is argued in the literature that the rich are more likely to utilize more healthcare services (like consultancy, bed, diagnostic tests etc.) [39] during their hospitalization and have longer stays in the public hospitals [45]. Moreover, most of the aged people in both the regions are not covered by any health insurance scheme (see Table 6 in Appendix). Consequently, the aged people are forced to spend the entire hospitalization expenses from their pocket. Hence, the IP utilization of the poorest class might be very low due to poor purchasing capacity [13].

Utilization of OP care and corresponding benefit incidence could be interpreted through access to free medicine from the healthcare facilities. Utilization of public facilities and benefit share are also high for the groups who have more access to free medicine in both the sectors (see Table 7 in Appendix). Surprisingly, more than 51% of the richest class in urban India also have access to free medicine and consequently the benefit share of the group is also the highest in the region. Importantly, the poorest class of the rural sector has the highest utilization of public OP care, share in total benefit and access to free medicine. Strengthening the public health facilities through National Health Mission (NHM) flexi-pool for NCDs, National Programme for Healthcare of the Elderly (NPHCE) might have helped the poorer sections of the society to access needed OP care in the rural sector. Analyzing state level data Selvaraj et al., (2010) [46] and Bose & Dutta (2018) [38] have showed that free distribution of medicine has impacted the health system in three ways – improvement in access to healthcare, financial risk protection and health system expansion. Therefore, it has to be noted here that following the changing disease pattern, regular updation of the essential drug list (EDL) is primarily needed. It would help the patients to get their required medicines from the public facilities. The study by Bose & Dutta (2018) [38] documented that in West Bengal most of the people are suffering from NCDs and most of them are utilizing public facilities for treatment. However, they are forced to purchase medicines from the market as most of the drugs listed in the EDL^10^ are either of communicable diseases or antibiotics.

Despite the nationally representative data has been used in this analysis, there are several limitations of the present study and the data. Following the literature, we have also considered the private OOPE as the proxy of actual cost of providing the services in the public facilities. On the other hand, health is a state subject. Therefore, the public health expenditure and OOPE largely varies across states. With some adjustment in estimation of private OOPE, we have tried to capture the state level variations in government health expenditure and OOPE. However, a proper costing study of the services provided through the public health system could give us a better picture. Additionally, the method applied to conduct the benefit incidence analysis needs substantial data points. However, given the limited sample size of the aged population suffering from NCDs and utilizing the IP and OP care, we have cross-classified the data into four MPCE groups. The results would have provided more information if the data could have been cross classified into quintile or deciles. Similarly, due to limited sample size we were forced to club the diseases into three broad groups. However, within NCDs, ailment nature, magnitude of illness and its impact on the patients might vary. To study the varying level of suffering within NCDs, more sample for each disease category (within NCDs) is needed. In the NSS data there is no specific information available on the severity of illness either for IP or OP care. Therefore, we have used the duration of stay in hospital or the duration of suffering from the illness as the proxy of severity of the illness. On the other hand, in NSS data, the OOPE for all the OP visits (for multiple visits within the reference period) are given together. It forces us to consider those individuals who have visited healthcare facilities for NCDs in all OP cases. Additionally, NSS is a self-reported data. The type of ailment a person is suffering from, type of facilities used, and related OOP expenditure are recorded based on the information provided by an individual. Therefore, there is potential chances of bias in the data. If NSS also provide the details of severity of illness and OOPE for each OP visit, estimation would be more robust. Sample size of the aged population. Following literature, we have used MPCE as a measure of wealth/socioeconomic status of a household.^11^ However, more relevant information like actual income or wealth status of the household could provide more clear picture of the economic position of a household. Finally, sample size of the UTs and the North-eastern states were very low. To analyze the data, we have clubbed all the UTs and all the North-eastern states to get enough sample for the study.

## Conclusions

Rapid ageing of the population and increasing NCDs among the elderly is one of the major public health challenges in India. Moreover, very high OOPE for the treatment even in the public facilities exacerbates the situation. To achieve the UHC goals, on the other hand, distribution of public subsidies should be effectively allocated among the socio-economically weaker sections. This analysis demonstrates that a substantial share of the public subsidies is still going to the richer sections for the treatment of NCDs among the elderly.

Analysis has also revealed that medicine is the most important component in OOPE during NCDs. Therefore, procuring medicine would be a policy-priority to reduce OOPE and increase utilization of healthcare facilities in the public sector. The policy makers in India should use the available information and monitor the extent of equity in public healthcare spending for NCDs among the elderly. As the share of elderly in the population and their suffering from NCDs are increasing, targeted policies should be taken to improve utilization, access to medicine, other healthcare services and public subsidy for the disadvantaged would be primarily important to achieve healthcare goals in India. Additionally, the utilization of the richer sections are more than any other groups. Therefore, an equal distribution of resources would benefit the richer sections disproportionately. To achieve the universal health coverage, focus should be given on treatment for all.

## Data Availability

The study is based on secondary data and all the data are available in the public domain. Specifically, the data and all other related information could be downloaded from Indian Council of Social Science Research (ICSSR) data service: Social Science Data Repository (available at - http://www.icssrdataservice.in/datarepository/index.php/catalog/107). Ministry of Statistics and Programme Implementation also provide unit level records at a nominal price (http://mospi.nic.in/sites/default/files/main_menu/data_discremination/ratelist_UnitData.pdf).

## Appendix

**Table 5:** MPCE Class wise Economic Dependence and Education of the Elderly in India (in %)

MPCE Class	Economic Dependence						Education							
	Rural			Urban			Rural				Urban			
	Not Dependent	Partially Dependent	Fully Dependent	Not Dependent	Partially Dependent	Fully Dependent	Illiterate	Up to Primary	Up to Secondary	Above Secondary	Illiterate	Up to Primary	Up to Secondary	Above Secondary
P	30.7	23.9	45.5	29.7	20.9	49.3	76.8	15.9	7.1	0.2	58.0	27.9	12.0	2.2
LM	23.3	24.7	51.9	29.2	15.7	55.1	71.5	18.5	8.0	2.0	42.6	25.4	21.1	10.9
UM	22.0	20.7	57.4	29.1	17.8	53.2	67.8	20.0	9.7	2.6	30.5	24.7	27.9	16.9
R	29.4	17.9	52.7	37.8	14.2	48.0	59.3	22.0	12.6	6.2	13.7	17.5	28.6	40.2
All	**26.7**	**21.5**	**51.9**	**31.9**	**16.9**	**51.2**	**68.2**	**19.3**	**9.6**	**3.0**	**33.6**	**23.3**	**23.4**	**19.7**

Source: Authors’ estimation based on NSS (2014)

**Table 6:** Distribution of Health Insurance Coverage of the Elderly in India (in %)

Type	Rural	Urban
Government Funded	16.73	14.86
Employer Supported & Others	0.88	7.23
Not Covered	82.39	77.90

Source: Authors’ estimation based on NSS (2014)

**Table 7:** Details of the Medicine Received during Treatment of NCDs in Public Facilities (in %)

MPCE Class	Inpatient								Outpatient							
	Rural				Urban				Rural				Urban			
	Not Received	Free	Partly Free	On Payment	Not Received	Free	Partly Free	On Payment	Not Received	Free	Partly Free	On Payment	Not Received	Free	Partly Free	On Payment
P	0.3	39.0	30.4	30.3	0.0	39.5	36.1	24.4	2.9	55.8	14.7	26.7	8.8	56.3	6.6	28.3
LM	0.0	36.8	37.2	26.0	0.7	37.1	35.3	26.9	5.8	30.0	23.2	41.0	17.6	52.0	9.3	21.1
UM	0.4	26.3	40.2	33.0	0.0	23.3	45.0	31.7	8.0	35.8	12.6	43.7	11.5	36.1	7.4	45.0
R	2.3	22.6	36.8	38.3	1.7	23.1	25.8	49.5	15.9	36.8	11.6	35.7	8.4	51.0	1.5	39.1
All	**0.8**	**30.6**	**36.4**	**32.2**	**0.6**	**30.5**	**35.6**	**33.4**	**7.2**	**41.8**	**15.4**	**35.6**	**11.3**	**49.6**	**6.3**	**32.8**

Source: Authors’ estimation based on NSS data, 2014

## Abbreviations

BIA: Benefit Incidence Analysis
CD: Communicable Diseases
EDL: Essential Drug List
FPMS: Fair Price Medicine Shop
FSU: First Stage Unit
IP: Inpatient
OP: Outpatient
LM: Lower Middle
LMICs: Low and Middle Income Countries
MPCE: Monthly Per Capita Expenditure Class
NCDs: Non-communicable Diseases
NHM: National Health Mission
NPHCE: National Programme for Healthcare of the Elderly
NSS: National Sample Survey
OD: Other Diseases
OECD: Organization for Economic Co-operation and Development
OOPE: Out-of-pocket Expenditure
P: Poorest
PPSWR: Probability Proportion to Size with Replacement
R: Richest
UFS: Urban Frame Survey
UHC: Universal Health Coverage
UM: Upper Middle
UN: United Nations
USU: Ultimate Stage Unit
UTs: Union Territories
WHO: World Health Organization

## Footnotes

1 The details methodology is available at NSS 71st round report: Social Consumption in India Health [25]

2 It is evident from National Health Accounts reports of India that the major contributor of total health spending is the OOP expenditure. And out-patient care has the maximum share in total OOP expenditure followed by the inpatient care. However, NSS collects information with two different recall period to minimize the bias in the data. Additionally, for out-patient care NSS reports the OOP expenditure for all the visits together within the reference period. Therefore, we study both the care separately to analyze the extent of OOP expenditure and benefit share.

3 The usual expenditure includes – usual monthly expenditure for household purposes, monthly average expenditure amount (dividing the total expenditure by 12) for durable goods purchased during last one year and approximate monthly values of wage in kind, home-grown stock and free stock.

4 Following the equivalence method suggest by Deaton (2003), we have also formed the MPCE classes and cross-checked the ranking of the MPCE groups for various indicators used in the paper [29]. However, there was no substantial change in the ranking of the MPCE classes in terms of utilization, OOP payment and benefit share.

5 The sample size for the North-eastern states and the UTs are very small in the NSS data. Therefore, we have clubbed all the North-eastern states (Sikkim, Nagaland, Mizoram, Tripura, Arunachal Pradesh and Meghalaya) and all the UTs (Chandigarh, Daman & Diu, Dadra & Nagar Haveli, Lakshadweep, Puducherry and Andaman & Nicobar Island) for the study.

6 Medical cost includes package, consultancy, medicine, diagnostic test, bed charge (only for inpatient), other medical expenses like attendant charges, physiotherapy, personal medical appliances, blood, oxygen, etc.

7 Horizontal equity: treating people with equal need equally; vertical equity: treating people with unequal need unequally.

8 Following Wagstaff (2012), we have replaced all the negative values with zero here [36].

9 It has to be mentioned here that to study the relative position and share in total subsidy benefit is the objective of the benefit incidence analysis. Estimation of actual benefit is not the purpose of the method.

10 The paper discusses about the Fair Price Medicine Shops (FPMS) of West Bengal. As per the contracts with the Government, the FPMS should at least keep all the medicines of the EDL and sell them at a discounted price to any patient carrying a valid prescription.

11 To measure the socioeconomic status of a household there are several indicators available in the literature. Many literature use wealth index or income as a measure of economic status of a household. However, NSS only reports monthly consumption expenditure of the households as a measure of economic indicator of a household.
